# Impact of orthogeriatric assessment on mortality in patients with periprosthetic hip fractures: a prospective study

**DOI:** 10.1007/s40520-026-03343-1

**Published:** 2026-02-16

**Authors:** Gianluca Canton, Andrea Marchetti, Andrea Sandrin, Belinda Trobec, Alex Buoite Stella, Paolo De Colle, Michela Zanetti, Luigi Murena, Chiara Ratti

**Affiliations:** 1 Orthopaedics and Traumatology Unit, Department of Medical, Surgical and health Sciences, Cattinara Hospital - ASUGI, Trieste University, Trieste, Italy; 2 Department of Medical, Surgical and Health Sciences, University of Trieste, via pascoli 31, Trieste, 34100 Italy; 3 Department of Medical, Surgical and Health Sciences, University of Trieste, Trieste, Italy; 4 Geriatric Clinic, Maggiore University Hospital, Azienda Sanitaria Universitaria Giuliano Isontina (ASUGI), Trieste, Italy; 5 School of Dietetics, University of Trieste - Pordenone branch, Pordenone, Italy

**Keywords:** Periprosthetic hip fracture, Mortality, Orthogeriatric assessment scores, MNA-SF, SPMSQ, ADL

## Abstract

**Background:**

Periprosthetic hip fractures (PPHF) are a serious and increasingly frequent complication of hip arthroplasty, associated with significant morbidity and mortality in older adults.

**Aims:**

To investigate the association between orthogeriatric assessment scores and six-month and one-year postoperative mortality in elderly patients undergoing surgery for PPHF.

**Methods:**

A prospective registry of patients aged ≥ 65 years treated surgically for PPHF at Trieste University Hospital was analysed. Clinical, radiographic, and perioperative data were collected. Orthogeriatric scores, including the Mini Nutritional Assessment – Short Form (MNA-SF), Short Portable Mental Status Questionnaire (SPMSQ), Activities of Daily Living (ADL), Charlson Comorbidity Index (CCI), Parker Mobility Index, and Nottingham Hip Fracture Score (NHFS), were recorded on admission and at follow-up. Univariate analyses were performed to identify predictors of six-month and one-year mortality.

**Results:**

Fifty-two patients (mean age 83.6 ± 8.1 years; 77% women) were included. The six-month and one-year mortality rates were 23.1% (12/52 patients) and 22.5% (9/40 patients), respectively. Higher mortality correlated significantly with poorer nutritional status (*MNA-SF*, *p* = 0.033; *p* = 0.011), lower cognitive performance (*SPMSQ*, *p* = 0.004, *p* = 0.002), reduced functional independence (*ADL*, *p* = 0.041, *p* = 0.026), and higher *Nottingham Hip Fracture Scores* (*NHFS*, *p* = 0.022, *p* = 0.047).

**Conclusions:**

In conclusion, orthogeriatric scores, particularly MNA-SF, SPMSQ, ADL, and NHFS, are strong predictors of mortality after PPHF.

## Introduction

As the population ages, there is a steady increase in prosthetic replacement procedures, accompanied by a parallel rise in complications related to these surgeries [1, 2]. Among the most serious complications there are periprosthetic fractures, with recent studies predicting their incidence to increase by 4.6% every ten years over the next three decades. Patients with periprosthetic fractures have a higher incidence of postoperative complications and an increased mortality compared with those who have undergone primary surgery. Despite gain in clinical experience and the numerous contributions in the scientific literature on this topic, the choice of optimal treatment strategies to maximise functional recovery while reducing the incidence of post-operative complications and mortality still remains a matter of debate [2, 3]. Moreover, older patients may have a worse prognosis due to poor bone quality and medical comorbidities [4]. In fact, the mortality after a periprosthetic fractures has been reported to be in the range of 2.1–10% in the first 30 days, and in that of 11% to 28% at 1 year [5, 6]. In addition, these patients often fail to regain full independence in the activities of daily living. As far as proximal femur fractures are concerned several geriatric assessment tools commonly used in the multidimensional evaluation of older adults have already been validated and are considered useful for evaluating patients comorbidities, including the Mini Nutritional Assessment – Short Form (MNA-SF) [7], the Short Portable Mental Status Questionnaire (SPMSQ) [8, 9], and the Activities of Daily Living (ADL) scale [10], assessed by the Modified Katz Index. These tools not only provide valuable insights into the patient’s baseline functional, nutritional, and cognitive status, but are also increasingly recognized for their prognostic value, helping to predict outcomes such as mortality, functional recovery, length of hospital stay, and the risk of postoperative complications [11].

The aim of the study was to assess the influence of nutritional, functional, and cognitive status at 6 months and 1 year mortality in elderly patients with periprosthetic hip fractures who underwent surgery.

## Materials and methods

### Data collection

A prospective registry was established to collect clinical and radiological data on patients undergoing surgery for PPHF. Institutional review board approval was obtained before initiation of the study.

The registry included all eligible patients treated at the Trieste University Hospital over a 15-month period, with an additional one-year follow-up for survival and functional outcomes. The objective of the registry was to evaluate the association between orthogeriatric clinical parameters and postoperative mortality. The inclusion criteria consisted of all individuals over 65 years of age who were admitted and underwent surgical treatment for a PPHF. Exclusion criteria included patients with associated injuries sustained during trauma (such as head, thoracic, or abdominal trauma, or fractures at other skeletal sites) that could influence mortality and functional recovery. Patients who declined participation or did not provide consent for data processing were excluded. Eligible individuals received detailed information regarding the study and provided written informed consent for data collection and analysis. The study was conducted in accordance with the Declaration of Helsinki and approved by the local ethics committee with protocol number 167_2022H.

Each patient underwent a comprehensive geriatric assessment upon admission and was subsequently followed by an orthogeriatric team throughout the entire care pathway. Several scales were administered to assess the patient’s general conditions: the Mini-Nutritional Assessment – Short Form (MNA-SF) [7], the Short Portable Mental Status Questionnaire (SPMSQ) [12], the Modified Katz Index of Activity of Daily Living (ADL) [13], the Charlson Comorbidity Index (CCI), the Parker Mobility Index [14] and the Nottingham Hip Fracture Score (NHFS). Radiological classification of the fracture was performed according to the Vancouver classification. Surgical and perioperative data were also recorded, including preoperative haemoglobin levels, time form admission to surgery, and type and duration of surgery. During hospitalization, complications such as delirium, acute renal failure, pneumonia, deep vein thrombosis, pulmonary embolism, and death were recorded. Follow-up assessments were performed at 6- and 12-months post-surgery and included re-evaluation of the clinical scores mentioned before. Mortality was assessed at each time point including only patients who had reached the respective follow-up milestone at the time of analysis. Radiological criteria were also assessed, including the grade of fracture consolidation according to the Tower and Beals classification criteria.

### Statistical analysis

A descriptive analysis of the variables involved was conducted, followed by a univariate analysis in order to assess the risk factors associated with mortality. For numerical variables with Gaussian distribution, the one-way ANOVA test was used. For numerical variables with a non-Gaussian distribution, non-parametric two-sample independent tests such as the Mann-Whitney test were performed. For categorical variables, the chi-square test was used. Univariate analysis was conducted by means of binary regression, expressing the relationship between a binary dependent variable and one or more independent variables by means of Odds Ratio. The confidence interval of the Odds Ratio was used to assess the significance of the association.

## Results

The study population included 52 patients who underwent surgery for PPHF, including 40 women and 12 men with an average age of 83.6 ± 8.1 years, ranging from a minimum of 65 to a maximum of 103 years. Population baseline characteristics are shown in Table 1. Upon admission, the average score of the MNA-SF was 11.87 ± 1.48, with a range from 8 to 14. The CCI had an average score of 5.37 ± 1.58, with a minimum of 2 and a maximum of 12. The NHFS had a mean of 5.08 ± 0.78, with scores ranging from 4 to 8. The pre-fracture Modified Katz ADL showed an average of 9.83 ± 2.04, with a minimum of 4 and a maximum of 12. The SPMSQ, in its simplified version, yielded a score of 0.56 ± 0.65, ranging from 0 to 2. The Parker Mobility Index prior to the fracture had a median of 6.29 ± 2.44, with scores between 0 and 9.

**Table 1:** Baseline characteristics of the study Population. Standard deviation (SD)

Variable	*n* (%)
Sex	52
Female	40 (76.9%)
Male	12 (23.1%)
Age (years) Mean ± SD / Range	83.6 ± 8. (Range: 65–103)
Vancouver Classification	
Type AL	1 (1.9%)
Type B1	21 (40.4%)
Type B2	23 (44.3%)
Type B3	1 (1.9%)
Type C	6 (11.5%)
Implant	
Arthroprothesis	40 (77%)
Endoprothesis	12 (23%)
Type	
Cemented	41 (79%)
Cementless	11 (21%)
Treatment	
Osteosynthesis	48 (92%)
Revisions	4 (8%)

**Table 2:** Univariate analysis of six-month mortality and one-year in periprosthetic fractures

Variables	Univariate OR 6 months	95% CI	*p*-value	Univariate OR 1 year	95% CI	*p*-value
Age	1,059	0,971-1,156	0,197	1,082	0,978-1,196	0,126
Sex	0,871	0,194-3,914	0,857	1,432	0,248-8,259	0,688
MNA-SF	0,614	0,393-0,961	0,033	0,469	0,261-0,842	0,011
SPMSQ	3,958	1,572-9,969	0,004	7,192	2,012–25,705	0,002
ADL	0,745	0,562-0,988	0,041	0,707	0,521-0,959	0,026
CCI	1,099	0,762-1,585	0,614	1,100	0,713-1,697	0,668
NHFS	2,498	1,142-5,465	0,022	2,410	1,014 − 5,731	0,047
Parker mobility score	0,831	0,645-1,069	0,150	0,779	0,589-1,032	0,081
Surgery duration (minutes)	0,958	0,958-1,000	0,045	0,953	0,920-0,988	0,009
Parker mobility index (follow-up)	0,493	0,274-0,886	0,018	0,462	0,215-0,991	0,047
MNA-SF (follow-up)	0,772	0,469-1,272	0,310	0,650	0,369-1,143	0,135
SPMSQ (follow-up)	2,580	0,923-7,214	0,071	4,631	1,225 − 17,508	0,024

Mini-Nutritional Assessment – short form (MNA-SF); short portable mental status questionnaire (SPMSQ); activity of daily living Katz score (ADL); the Charlson comorbidity index (CCI); Nottingham hip fracture score (NHFS). Odd risk (OR), confidence interval (CI)

Fractures were classified according to the Vancouver classification as AL in one case (1,9%), B1 in 21 cases (40,4%), B2 in 23 cases (44,3%), B3 in one case (1,9%) and C in 6 cases (11,5%).

Regarding the preoperative waiting time, only four out of 52 patients underwent surgery within the first 24 h of admission, 12 between 24 and 48 h, 16 before 72 h, and 20 after 72 h.

**Fig. 1:**
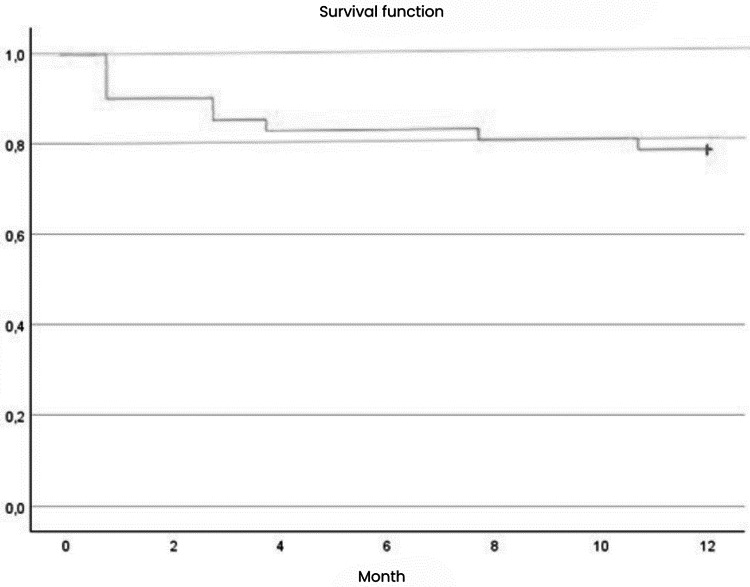
One-year survival curve according to Kaplan-Meier

Mortality was assessed over a six-month period for the entire sample of 52 patients and over a one-year period for 40 patients. In the first six months, 12 deaths occurred out of 52 patients (23.1%), with four in the immediate postoperative period (33,3%), four in the second month (33,3%), two in the third month (16,7%), one in the seventh month (8,3%), and one in the tenth month (8.3%). Over the course of the year, nine deaths were recorded out of 40 patients (22.5%). Figure 1 shows the one-year survival curve according to Kaplan-Meier.

At the one-year follow-up, the average MNA-SF score was 11.88, the SPMSQ showed a median of 0.46, ranging from 0 to 2, and the Parker Mobility Index averaged 4.16, with a range from 0 to 9.

The univariate analysis showed a significant association between the previous situation according to the comprehensive geriatric assessment and mortality. However, due to the limited sample size, these factors were not validated through multivariate analysis. In detail, the following factors significantly influenced mortality at six months and one year: poor nutritional status, cognitive impairment, functional impairment, high risk according to NHFS. Additionally, at one-year, preoperative haemoglobin levels and the duration of surgery emerged as relevant factors. Table 2 show the univariate analysis of 6 months and 1 year mortality.

**Fig. 2:**
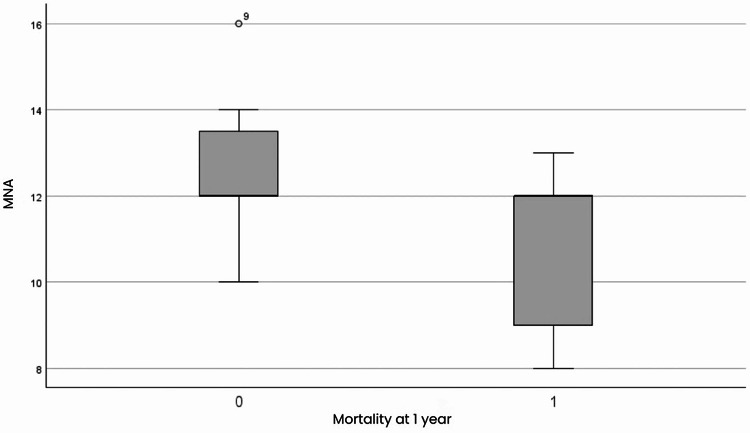
Mini Nutritional Assessment – Short Form based on mortality at one year. Group 0: subjects alive at one year; Group 1: subjects deceased at one year

The MNA-SF score showed a correlation with mortality at both six months and one year (Fig. 2), indicating that patients with better nutritional status had a lower risk of death with an OR of 0.614 (95% CI: 0.393–0.961) at six months and 0.469 (95% CI: 0.261–0.842) at one year. Patients with a better nutritional status had a reduction in mortality risk of 53.1% at one year and 38.6% at six months (*P* = 0.011 and *P* = 0.033, respectively). For every unit increase in MNA-SF score, mortality odds ratio is reduced by 53.1%.

The SPMSQ score showed a strong association between cognitive impairment and a significantly increased risk of mortality, with an OR of 3.958 (95% CI: 1.572–9.969) at six month and 7.192 (95% CI: 2.012–25.705) at one year. At six months, individuals with lower SPMSQ scores had a 2.95-fold higher risk of death compared to those with better cognitive function (*P* = 0.004), and this risk increased to 6.19-fold at one year (*P* = 0.002).

The Modified Katz score of ADLs also proved to be a determinant of mortality, with greater independence in daily activities associated with a significantly lower risk with an OR of 0.745 (95% CI: 0.562–0.988) at six months and 0.707 (95% CI: 0.521–0.959) at one year. The most independent subjects had a risk reduction of 29.3% at one year and 25.5% at six months (*P* = 0.026 and *P* = 0.041, respectively).

**Fig. 3:**
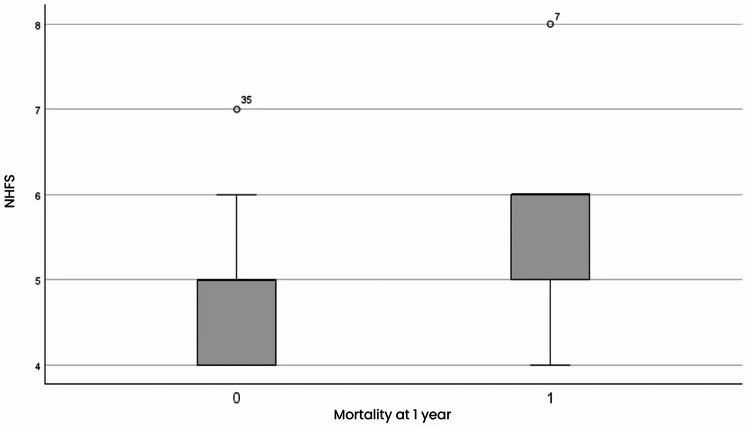
Nottingham hip fracture score versus mortality at one year. Group 0: subjects alive at one year; group 1: subjects dead at one year

The NHFS score correlated with mortality (Fig. 3); higher NHFS scores were associated with a significant risk of mortality, with an OR of 2.498 (95% CI: 1.142–5.465) at six months and 2.410 (95% CI: 1.014–5.731) at one year. Patients with higher NHFS scores had a 2.41-fold increased risk of mortality compared to the group with the lowest NHFS score, with a 141% increased risk at one year and 150% increased risk at six months (*P* = 0.047 at one year and *P* = 0.022 at six months).

Complications during hospitalization were found to be strongly correlated with the risk of mortality at six months with an OR of 13.571 (95% CI: 2.183–84.372), although this association was not confirmed at one year (OR: 4.143, 95% CI: 0.494–34.746, p-value 0.190). Preoperative haemoglobin levels were also identified as a factor influencing mortality at one year, with a decreased level correlating with an increased risk (OR = 0.462, CI: 0.232–0.918). Furthermore, the duration of surgery exhibited a slight association with one-year mortality; longer surgery duration was slightly correlated with increased mortality, with an OR of 0.953 (95% CI: 0.920–0.988). However, these two correlations were not confirmed for mortality at six months.

Finally, the Parker Mobility Index at postoperative follow-up was also correlated with mortality, showing a lower risk of high mortality with an OR of 0.493 (95% CI: 0.274–0.886) at six months and 0.462 (95% CI: 0.215–0.991) at one year for subjects who had regained motor skills. In fact, Parker’s Mobility Index showed that subjects with greater walking ability at home, outdoors and in social activities at control showed a highly significant reduction in mortality risk, 53.8% at one year and 50.7% at six months (*P* = 0.047 and *P* = 0.018, respectively).

Regarding time to surgery, no difference in terms of one year mortality was observed between subjects who underwent surgery within two days of the injury (OR 1,875, 95% CI 0,396-8,875, p-value = 0,428) and those who underwent surgery after three days (OR 1,035, 95% CI 0.280–3.826, p-value 0,959). Regarding age, no significant correlations with mortality were found. Similarly, sex did not reveal significant differences, despite the predominance of female subjects, likely due to greater longevity and a higher incidence of fractures from bone quality. Additionally, no significant associations were identified between mortality and the following parameters: CCI score, preoperative complications, or haemoglobin levels during the first five postoperative days.

## Discussion

The present study highlights the prognostic significance of orthogeriatric assessment tools in predicting mortality in patients undergoing surgery for PPHF. In particular, nutritional status, cognitive status, dependence in ADL, and risk as measured by NHFS are significantly associated with both six-month and one-year mortality. In contrast, age, sex, and the CCI did not show a significant correlation with survival, in contrast with some assumptions reported in the literature.

The observed one-year mortality rate of 22.5% in our cohort aligns with previously reported values in the literature, which range from 13% to 30% depending on study design and population characteristics [15]. However, our mortality rate exceeds the 13.4% reported in the meta-analysis by Lamb et al. [16], which included 35 cohort studies [16]. This discrepancy may be explained by differences in patient demographics, inclusion criteria, and methodological approaches. Notably, Lamb et al. highlighted that mortality following periprosthetic fractures is influenced by factors such as advanced age, the presence of multiple comorbidities, and a high rate of reoperations [17, 18]. The six-month mortality rate of 23.1% observed in our study underscores the particularly high risk associated with the early postoperative period, during which approximately one-third of all deaths occurred.

Contrary to what has been reported in the literature, our study found that the CCI does not have a significant influence on mortality. In fact, Myers et al. [19] previously reported that higher CCI scores correlated with increased mortality in older patients with distal femur fractures. The lack of association in our cohort may be due to the homogeneity of high comorbidity scores across the sample, which limited discriminative power [19].

Conversely, the NHFS proved to be a reliable predictor of mortality at both six months and one year, with an odds ratio (OR) of 2.498 and 2.410 respectively. These findings are supported by Grewal et al. (2021), that demonstrated a strong correlation between one year mortality and NHFS in PPHF patients. Given its simplicity and predictive accuracy, NHFS can be considered a valuable preoperative screening tool for risk stratification [20, 21].

The MNA-SF emerged as a significant predictor of mortality. Patients with a better nutritional status had a reduction in mortality risk of 53.1% at one year and 38.6% at six months. For every unit increase in MNA-SF score, mortality odds are reduced by 53.1%. This is a result not previously reported in the literature for PPHFs, which suggests the importance of assessing the nutritional status of patients prior to surgery and monitoring it over time by ensuring adequate nutrition, as already demonstrated for proximal femur fracture patients [10].

Similarly, cognitive impairment, measured by the SPMSQ, was strongly associated with increased mortality. Patients with lower SPMSQ scores had a 2.95-fold increased risk of death at six months and a 6.19-fold increase at one year. This previously unreported finding indicates that patients with cognitive impairment are at a significantly higher risk of mortality. It underscores the importance of cognitive assessment and the management of cognitive dysfunction in clinical practice to improve survival outcomes [22].

Functional status, assessed using the modified Katz ADL scale and the Parker Mobility Index, also showed a strong relationship with survival. Greater independence in ADLs was associated with a 29.3% reduction in one-year mortality and a 25.5% reduction at six months. To the best of our knowledge, no articles or studies were found reporting a similar result. Although early weight-bearing did not yield significant results, early patient mobilization and the recovery of independence in activities of daily living proved to be of primary importance.

In fact, Parker’s Mobility Index showed that subjects with greater walking ability in the home, outdoor and in social activities at control showed a highly significant reduction in mortality risk (53.8% at one year and 50.7% at six months). This indicates that reduced functional status and impaired mobility are significant risk factors in terms of survival, with individuals more vulnerable to disease- or intervention-related complications, resulting in an increased risk of mortality [23].

As far as time to surgery is concerned, no difference in mortality was observed between subjects operated on within two days of injury and those who underwent surgery after three days. This finding results in line with other literature reports by Smolle et al. [18] and Bliemel et al. [24]. These studies showed that time to surgery is not significantly associated with an increased risk of complications and mortality, nor does it lead to a reduction in mortality rates in the acute or mid-term phase. In fact, in most cases, surgical delay is clinically justified, as patients often need to be medically stabilized before undergoing surgery. However, the goal should be early surgical treatment to achieve early mobilisation and avoid non-surgical secondary complications [18, 24].

Strengths of the study: demonstrating that nutritional status, cognitive function, prior functional status, and risk as measured by NHFS are predictors of mortality (or survival) in patients with PPHF.

The present study limitations include the sample size and the relatively low number of death events, which limited a comprehensive assessment of the impact of each factor on the risk of death by a multivariate analysis. Furthermore, due to the small sample size, the results may not be generalizable to other populations. Increasing the sample size and the number of events would enable a more accurate determination of the true impact of each factor.

## Conclusions

In conclusion, orthogeriatric scores, particularly MNA-SF, SPMSQ, ADL, and NHFS, are strong predictors of mortality after PPHF. Further studies are needed to confirm whether integrating structured orthogeriatric and physiatric evaluation into perioperative care can improve risk stratification, guide personalized management, and ultimately enhance survival and functional recovery.

## Data Availability

No datasets were generated or analysed during the current study.
